# TECHNOLOGY-ENHANCED PSYCHOTHERAPY IMPROVES LIFE SATISFACTION AMONG OLDER ADULT VETERANS IN LONG-TERM CARE

**DOI:** 10.1093/geroni/igz038.1863

**Published:** 2019-11-08

**Authors:** Jonathan Sills, James A Mazzone, Flora Ma, Peter Louras, Erickson Alexander

**Affiliations:** 1 Veterans Affairs Palo Alto Health Care System, Palo Alto, California, United States; 2 Veterans Affairs Palo Alto Health Care System, Menlo Park, California, United States; 3 Palo Alto University, Palo Alto, California, United States

## Abstract

To buffer the risk of declining life satisfaction among a Veteran cohort residing within a Veteran’s Affairs long term care facility, a new model of care called Individualized Non-Pharmacological Services Integrating Geriatric Health and Technology (INSIGHT) therapy was developed and evaluated. Consistent with the INSIGHT therapy model, traditional psychotherapy interventions including reminiscence, behavioral activation, and relaxation exercises were modified such that they could be delivered on a digital platform. A paired sample T-test was performed to identify the effects INSIGHT Therapy had on Veteran satisfaction with life. Findings indicated that Veteran life satisfaction ratings the month prior (M= 19.6522) to the initiation of INSIGHT intervention and the month following three months of INSIGHT intervention (M=22.4783) show that the satisfaction with life increased among residents (t(22)=-2.334, p=.028). Effect size = 0.489. These results suggest that INSIGHT therapy interventions help to contribute to an increase in life satisfaction among an older adult Veteran cohort residing within a Veteran’s Affairs long term care facility.

